# Exercise Induced Worsening of Mechanical Heterogeneity and Diastolic Impairment in Long QT Syndrome

**DOI:** 10.3390/jcm10010037

**Published:** 2020-12-24

**Authors:** Dafni Charisopoulou, George Koulaouzidis, Lucy F. Law, Annika Rydberg, Michael Y. Henein

**Affiliations:** 1 Institute of Public Health and Clinical Medicine, Umea University, 90187 Umea, Sweden; koulaou@yahoo.co.uk (G.K.); michael.henein@umu.se (M.Y.H.); 2 Division of Pediatric Cardiology, Department of Pediatrics, Amalia Children’s Hospital, Radboud University Medical Center, 6500 HB Nijmegen, The Netherlands; 3 Academic Centre for Congenital Heart Disease, 6500 HB Nijmegen, The Netherlands; 4 Department of Clinical Sciences, Paediatrics, Umea University, 90187 Umea, Sweden; lucy.law@umu.se (L.F.L.); annika.rydberg@umu.se (A.R.)

**Keywords:** long QT syndrome, exercise stress echocardiogram, mechanical dispersion, diastolic myocardial function, exercise

## Abstract

Background: Electromechanical heterogeneities due to marked dispersion of ventricular repolarisation and mechanical function have been associated with symptoms in long QT syndrome (LQTS) patients; Aim: To examine the exercise response of longitudinal LV systolic and diastolic myocardial function and synchronicity in LQTS patients and its relationship with symptoms; Methods: Forty seven (age 45 ± 15 yrs, 25 female, 20 symptomatic) LQTS patients and 35 healthy individuals underwent an exercise test (Bruce protocol). ECG and echo parameters were recorded at rest, peak exercise (p.e.), and recovery; Results: LQTS patients had prolonged and markedly dispersed myocardial contraction, delayed early relaxation phase, and significantly decreased filling time at all exercise phases. Unlike controls, these electromechanical disturbances deteriorated further with exercise, during which additional decrease of the LV diastolic myocardial function and attenuated LV stroke volume were noted. Such abnormal responses to exercise were seen to a greater degree in symptomatic patients and in the LQT1 subgroup and improved with B-blocker therapy. Worsening myocardial contraction dispersion at p.e. was the strongest discriminator for previous clinical events, and its discriminating power excelled further by adding early relaxation delay; Conclusions: Electromechanical disturbances were shown to worsen during exercise in LQTS patients and were more pronounced in those with previous arrhythmic events.

## 1. Introduction

One of the greatest challenges in cardiology practice remains the prompt identification of long QT syndrome (LQTS) mutation carriers who are at highest risk of developing adverse cardiac events such as arrhythmias, syncope, or sudden cardiac death [1]. This has been particularly difficult in cases where the patients have a normal QTc interval or no previous symptoms [2]. Inherited LQTS was initially thought to be solely an electrical phenomenon with prolonged action potential and exaggerated dispersion of spatiotemporal repolarisation [3]. However, such electric abnormalities have been shown to correspond to mechanical ones, referred to as electromechanical (EM) disturbances [4,5]. Indeed, prolonged myocardial contraction and enhanced regional and transmural mechanical dispersion have been reported at rest in LQTS, particularly in symptomatic patients [6,7]. However, mechanical dispersion is shown, in other conditions, to be associated with impaired myocardial diastolic function [8]. Recently, resting subclinical diastolic disturbances and shortened LV filling duration have been described in LQTS [9,10]. Moreover, EM coupling disturbances in the form of reversed (negative) EM windows have been shown to worsen during stress in LQTS subjects [4].

To investigate the combined effect of prolonged myocardial contraction and increased mechanical dispersion on myocardial diastolic function and symptoms in LQTS, we used two-dimensional speckle-tracking echocardiography. We adopted this approach to examine the exercise response of longitudinal LV systolic and diastolic myocardial function and synchronicity in a group of LQTS patients, in an attempt to identify high risk carriers who have had previous cardiac events.

## 2. Methods

### 2.1. Study Population

The study group included 47 genetically confirmed LQTS patients, who have been regularly followed-up with at the Umeå University Hospital. The control group consisted of 37 age- and gender-matched healthy individuals, selected from a local database of healthy people who were not on any medical treatment. Subjects with evidence of ischaemic heart disease or at high risk of atherosclerosis were not included in the study. LQTS patients were categorized as asymptomatic or if there was documented history of cardiac events such as syncope, cardiac arrest, and ventricular tachyarrhythmias, as symptomatic. Two independent operators blinded to subjects’ clinical and demographic details performed the measurements and analysed the studied parameters.

### 2.2. Exercise Protocol

All participating subjects underwent a bicycle exercise test in a semi-supine position (slightly left lateral tilt) using a General Electric—GE ergometer (model 900, Ergoline GmbH, Bitz, Germany) with an increasing workload of 10 Watts every 2 min. Arterial blood pressure (BP), ECG, and full Doppler-echocardiogram were recorded and analysed in three phases: (1) at rest, just before exercise; (2) at peak exercise (p.e.), when 85% of the maximum predicted heart rate (HR) for age was achieved; and (3) at 4 min into recovery.

### 2.3. The Electrocardiogram (ECG)

A 12-lead ECG was continuously recorded, throughout exercise, at 25 mm/s speed using a conventional electrocardiograph. At the end of each of the three study phases, manual measurements of RR, QT, and QRS intervals were made. The QRS duration was measured from the onset of the QRS complex to its end. The QT interval was measured from the start of the QRS to the end of the T-wave, at the intersection point of the descending limb of the T-wave with the isoelectric line. QT measurements were corrected for HR using the Bazett formula (QTc = QT/√RR).

### 2.4. Echocardiography

Two-dimensional echocardiograms were performed in the semi-supine position using a Vivid 7 echocardiograph (General Electric (GE), Horten, Norway) and interfaced to an adult 1.5–4.3 MHz phased array transducer. At the end of each exercise stage, digital loops of three cardiac cycles were consecutively acquired from the standard apical four- and two-chamber and parasternal long- and short-axis views. ECG (lead II) was superimposed on and acquired from all 2D and Doppler recordings. The analysis was blindly performed offline using a dedicated workstation (EchoPAC, version 8.0.1; GE, Waukesha, WI, USA).

#### 2.4.1. Conventional and Doppler Echocardiography

The Simpson’s biplane method was used to estimate the LV ejection fraction. LV stroke volume (SV) was estimated as the product of the LV outflow velocity time integral (VTI) and cross-sectional area [11]. LV filling velocities (E and A waves) were recorded from the apical four chamber [11]. Time to peak early LV filling (tE) was defined by the time interval from the QRS onset on the superimposed ECG to the onset of the E wave on the pulsed-wave (PW) Doppler recording. LV filling time (FT) was measured from the onset of the E-wave to the end of the A-wave velocity and was expressed as a percentage of the corresponding cardiac cycle duration (RR interval). LV diastolic function and LV filling pressures were assessed based on the recommendations of the European and American society of Echocardiography [11].

#### 2.4.2. LV Two-Dimensional Speckle-Tracking Echocardiography (STE)

**Figure 1:**
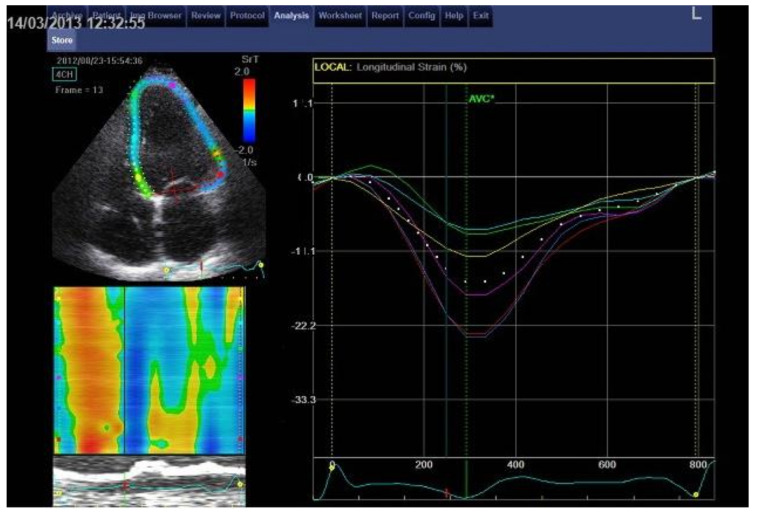
Speckle-tracking 2D-strain imaging (apical 4-chamber view). On the selected optimal image, the left ventricular (LV) endocardial border is manually traced on the end-diastolic frame and the software automatically draws the epicardial contour (left upper figure). The accuracy of the automated tracking of the cardiac outlines is assessed visually and is adjusted manually as required so that the region of interest (ROI) fits the LV wall thickness. ROIs are then subdivided into the standard segments (left upper figure). Thereupon longitudinal global strain (white dotted curve) and regional longitudinal strain curves (separate, coloured curves for each of the left ventricular wall segments seen in this view) are generated by the software (right figure).

**Figure 2:**
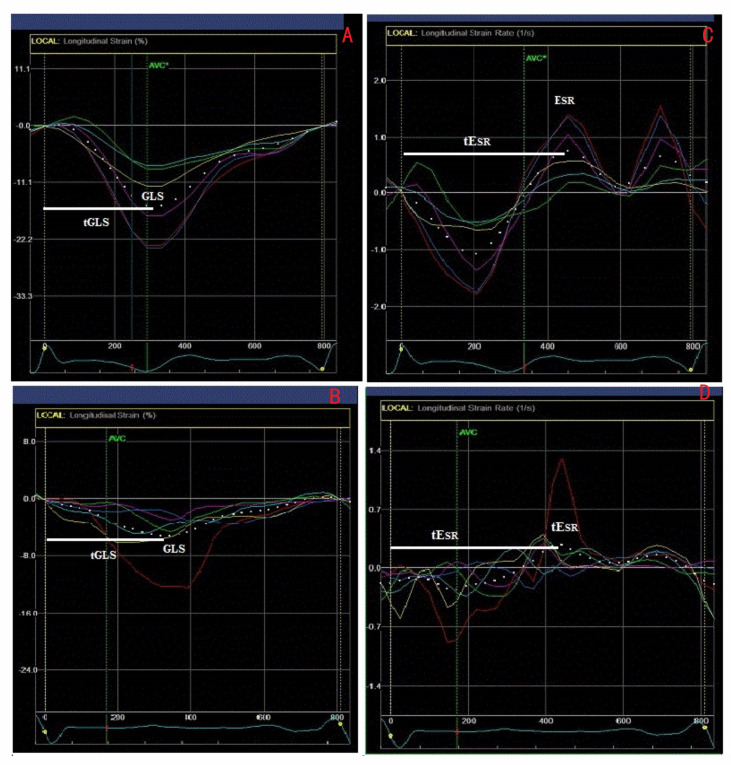
Left ventricular (LV) myocardial strain (**A**,**B**) and strain rate (**C**,**D**) curves in a healthy participant (**A**,**C**) and in a LQTS (**B**,**D**) patient. In the LQTS patient there is LV dyssynchrony and prolonged duration of global LV (white dotted curve) myocardial shortening (tGLS) with peak LV global longitudinal strain (GLS) occurring after the aortic valve closure (AVC); also peak global early diastolic strain rate (tESR) occurs later.

LV longitudinal systolic and early diastolic strain (S) and strain rate (SR) assessment by STE was performed at basal, mid, and apical levels (16 LV segments) from parasternal long axis and apical four- and two-chamber views [12]. On the selected optimal left heart image, the LV endocardial border was manually traced on the end-diastolic frame and the software automatically drew the epicardial contour (Figure 1). The region of interest (ROI) was automatically divided by the software into six standard segments for the LV and three slices (basal, mid-ventricle, and apical) for the LV free wall and the interventricular septum. Mean S and SR curves were generated for each ROI by the software (Figure 1). One LQT and two control subjects, in whom not all 16 segments could be assessed due to high body mass index, were not included in the study. As a result, we included 47 LQTS patients and 37 controls in our study. LV global longitudinal systolic strain (GLS) and global early diastolic strain rate (E_SR_) were defined as the 16 LV segments’ average value of peak longitudinal negative systolic S and peak positive early-diastolic SR, respectively (Figure 2).

For the time-to-peak systolic LV longitudinal strain (tGLS), we measured the time interval from the QRS onset on the superimposed ECG to peak negative systolic LV longitudinal S including any post-systolic contractions (Figure 1). The time to LV longitudinal early diastolic SR (tE_SR_) was similarly measured from the QRS onset to peak positive early-diastolic SR (Figure 1). The tGLS and tE_SR_ intervals were measured from each of the 16 segmental S and SR curves and their average was calculated. All time intervals were expressed as proportion of the R-R interval (%). LV longitudinal contraction dyssynchrony (SD-tGLS) was assessed by the standard deviation of tGLS intervals derived from the 16 LV segments. Early diastolic temporal discordance (E-E_SR_), as a reflection of potential discoordination between early LV ventricular filling and myocardial relaxation, was assessed by the time difference between tE and tE_SR_ [12]. Percentage changes (Δ,%) from baseline to p.e. and recovery were calculated.

### 2.5. Statistical Analysis

Statistical analyses were made using the Statistical Package for the Social Sciences (SPSS) for Windows (version 13.0, SPSS Inc., Chicago, IL, USA). A two-tailed *p*-value < 0.05 was considered a significant difference. Continuous variables were expressed as mean ± standard deviation (SD) and categorical variables as absolute number and percentage (%). The Chi-square test was used to assess if distributions of categorical variables differed from one another. To compare groups, we used the Student’s *t* test for variables with normal distribution and Mann–Whitney *U* test for non-normally distributed variables. Correlations were assessed with Pearson’s test. The sensitivity and specificity of QTc, tGLS, tE_SR_, and SD-tGLS for identifying previous clinical events in LQTS patients were investigated using the receiver operating characteristic (ROC) analysis. Inter-and intra-observer variability were assessed by calculating Cronbach’s alpha reliability coefficient in 10 randomly selected patients at the exercise phases.

### 2.6. Ethics Approval

The study protocol followed the ethical guidelines of the 1975 Declaration of Helsinki and was approved by the Regional Ethics Review Board, Umeå, Sweden; Dnr 2011-339-31M. Informed participation consent was obtained from all subjects.

## 3. Results

### 3.1. Baseline Characteristics

The study group consisted of 47 LQTS mutation carriers (36 LQT1 and 11 LQT2) who were compared with 35 healthy controls, matched for age (45 ± 15 vs. 47 ± 13 years, *p* = 0.2) and gender (53 vs. 54% females, *p* = 0.3). LV EF was not different between LQTS and controls (65 ± 6 vs. 67 ± 7%, *p* = 0.3). No ECG or echocardiographic disturbances indicative of ischaemic heart disease were detected in any of the participants. In addition, no evidence for bundle branch block was observed on the ECG. Among the LQTS subjects, 20 (43%) were symptomatic based on documented history of syncope, cardiac arrest, or arrhythmia; three had an implantable cardioverter-defibrillator (ICD) inserted. Among the LQTS patients, 14 (70%) of the symptomatic and 11 (41%) of the asymptomatic patients were receiving B-blocker therapy.

### 3.2. Exercise Response

#### ECG and Symptoms During Exercise

Arrhythmia during the exercise test appeared only in two of the symptomatic carriers, who developed frequent ventricular extra-systoles when close to reaching 85% of the maximum predicted HR. The rest of the participants did not develop any symptoms; the exercise test was terminated because of achieving target HR or fatigue. There was no significant difference in maximum achieved workload between patients and controls (125 ± 26 vs. 131 ± 35 Watts, *p* = 0.3). Maximum HR at three phases (rest: 69 ± 10 vs. 68 ± 10 bpm, *p* = 0.8; p.e.: 121 ± 17 vs. 120 ± 15 bpm, *p* = 0.7; recovery: 69 ± 10 vs. 68 ± 9 bpm, *p* = 0.7) was not different between the two groups. Similarly, there were no differences in systolic BP (rest: 117 ± 12 vs. 119 ± 13 mmHg, *p* = 0.8; p.e.: 176 ± 18 vs. 174 ± 14 mmHg, *p* = 0.5; recovery: 115 ± 15 vs. 118 ± 13 mmHg, *p* = 0.5).

### 3.3. Electromechanical Response to Exercise

#### 3.3.1. QTc

The QTc interval was longer in LQTS patients than in controls at the three phases (*p* < 0.0001). It prolonged further at p.e. in patients in contrast to controls, in whom it shortened (ΔQTc: +10 ± 9 vs. −5.5 ± 3.8%, *p* < 0.0001). At recovery, QTc remained longer with respect to baseline in patients but reached baseline values in controls (ΔQTc: +6.2 ± 5 vs. + 0.007 ± 2%, *p* < 0.0001).

#### 3.3.2. LV Longitudinal Contraction Function

There were no differences in LV EF at all phases between LQTS subjects and controls (*p* = 0.9, Table 1). Patients tended to have lower GLS at all phases (*p* < 0.005, Table 1). The extent of GLS increase with exercise was less in the patients than in controls (ΔGLS: +12 ± 32 vs. +25 ± 16%, *p* < 0.0001). On the other hand, tGLS was longer in patients compared to that of controls at all phases. The tGLS prolonged from baseline to p.e. in patients but shortened in controls (ΔtGLS: +26 ± 18 vs. −4.6 ± 3%, *p* < 0.0001) and remained longer into recovery in relation to baseline (ΔtGLS: + 6 ± 9 vs. −5 ± 4%, *p* < 0.0001) only in patients.

#### 3.3.3. LV Longitudinal Diastolic Function

Longitudinal E_SR_ was significantly lower in LQTS carriers than in controls at all phases (Table 1) and the magnitude of its increase with stress was smaller in patients (ΔE_SR_: +20 ± 32 vs. +51 ± 35%, *p* = 0.0001). Higher LV filling pressure in patients was also suggested by E/e’ and E DT (Table 1).

Early diastolic relaxation (tE_SR_) was delayed at all phases (Table 1) in LQTS subjects in whom the extent of this delay increased significantly from rest to p.e. (ΔtE_SR:_ +15 ± 14 vs. −4 ± 5%, *p* < 0.0001). At recovery and in contrast to that of controls, tE_SR_ remained delayed in patients but less than it was during stress (ΔtE_SR_: +6 ± 10 vs. −2 ± 6%, *p* < 0.0001). FT was shorter in patients in the three phases (Table 1) and shortened significantly at p.e., in contrast to its behaviour in controls (ΔFT: −23 ± 10 vs. 2 ± 3%, *p* < 0.0001); this was seen to a lesser extent during recovery (ΔFT: −8 ± 3 vs. −1 ± 2%, *p* < 0.0001).

#### 3.3.4. LV Mechanical Dyssynchrony

LQTS carriers had pronounced dispersion of LV myocardial contraction (SD-tGLS) at all phases (Table 1). Opposite to controls, SD-tGLS increased at p.e. and recovery with respect to baseline (ΔSD-tGLS: +5 ± 15 vs. −23 ± 20% and +5 ± 16 vs. −12 ± 28%, *p* < 0.0001). In addition, early diastolic myocardial SR velocity was delayed with respect to peak LV inflow velocity [Δt(E-E_SR_)] (Table 1).

#### 3.3.5. Stroke Volume

The stroke volume was lower in LQTS patients than in controls only at p.e. (Table 1). Moreover, the exercise increase of SV was significantly lower in patients (Δ SV: +2 ± 1 vs. 32 ± 4%, *p* < 0.0001).

### 3.4. Electromechanical Correlations

QTc correlated modestly with SD-tGLS (r = 0.45, *p* = 0.001), which showed positive correlation with tGLS (r = 0.6, *p* < 0.0001) and tE_SR_ (r = 0.57, *p* < 0.0001). However, prolonged tE_SR_ correlated with reduced FT (r = −0.41, *p* < 0.0001), which in turn correlated with lower SV (r = 0.27, *p* = 0.001).

### 3.5. Symptomatic vs. Asymptomatic LQTS Patients

LV GLS and E_SR_ were significantly lower at all phases in symptomatic compared to asymptomatic patients (Table 2). In addition, tGLS, tE_SR_, and t (E-E_SR_) intervals were longer and SD-tGLS of a greater degree in symptomatic patients (Table 2). Finally, in symptomatic patients, LV FT was shorter at rest and peak stress and SV response to exercise was more attenuated (Table 2). The maximal achieved workload was not different between symptomatic and asymptomatic patients (123 ± 24 vs. 127 ± 27 Watt, *p* = 0.8)).

#### Discriminating Previous Clinical Events

Mechanical dispersion (SD-tGLS) at p.e. was the strongest discriminator of previous clinical events (AUC 0.960, 95% CI: 0.020–0.995, *p* < 0.0001), stronger than QTc (AUC 0.569, 95% CI: 0.402–0.735). The combination of tE_SR_ and SD-tGLS improved this discriminator ability (AUC 0.970, 95% CI: 0.015–0.997).

### 3.6. Treatment with B-Blockers

**Figure 3:**
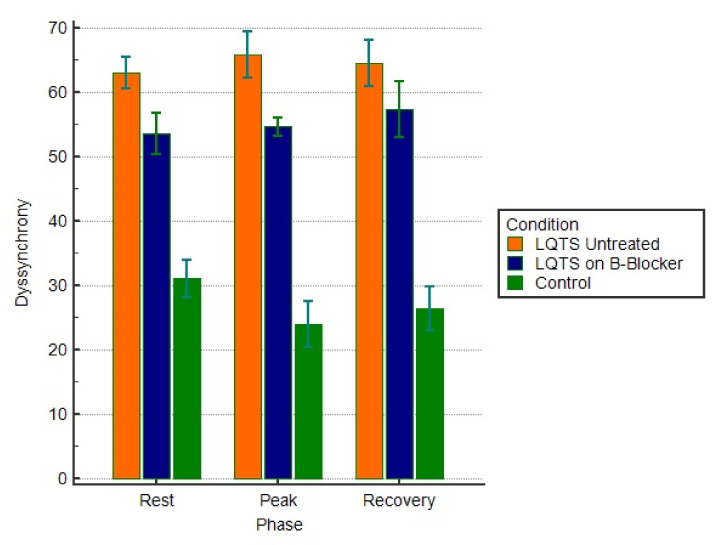
Dyssynchrony (SD-TGLS) response to exercise in LQTS mutation carriers treated with B-blockers, untreated LQTS mutation carriers, and controls *(p* < 0.01).

QTc did not differ between patients treated or untreated with B-blockers at rest, p.e., or recovery (Table 3). However, at rest and at recovery patients on B-blockers had higher GLS and E_SR_, shorter tGLS and tE_SR_, less pronounced SD-TGLS, and longer FT (Table 3). These differences were more pronounced at p.e. with patients on B-blocker therapy showing higher GLS and E_SR_, less prolonged contraction duration, smaller degree of dyssynchrony, early relaxation delay, and longer FT (Table 3, Figure 3). Although SV at rest was not different between the two subgroups, it was lower at p.e. and recovery in patients not taking B-blockers (Table 3).

### 3.7. Exercise Response According to LQTs Genotype

During exercise, LQT1 patients showed longer QTc (503 ± 47 vs. 458 ± 41, *p* = 0.03), greater SD-tGLS (62 ± 8 vs. 56 ± 4, *p* = 0.02), and longer tE_SR_ (71 ± 5 vs. 66 ± 7, *p* = 0.01) than those of LQT2 patients. However, at rest or during recovery, these parameters were not different between the two genotype subgroups (*p* > 0.3).

At peak stress, the QTc prolongation was greater in LQT1 than in LQT2 patients (ΔQTc: +12 ± 8 vs. −5.5 ± 3.8 %, *p* < 0.0001) but not different during recovery (ΔQTc: +7 ± 5 vs. +8 ± 2%, *p* = 0.5). Additionally, at p.e., mechanical contraction increased in LQT1 but remained almost unchanged in LQT2 patients (ΔSD-GLS: +6 ± 2 vs. −1 ± 12%, *p* < 0.0001), whereas it increased more in LQT2 patients during recovery (ΔSD-tGLS: +6 ± 4 vs. +11 ± 9%, *p* = 0.001). The degree of tGLS and tE_SR_ prolongation at p.e. and recovery was similar for the two groups (*p* > 0.5).

FT shortening was also greater at p.e. in LQT1 than that of LQT2 patients (ΔFT: −27 ± 11 vs. −10 ± 8%, *p* < 0.0001) but of similar magnitude during recovery (ΔFT: −10 ± 4 vs. −9 ± 3%, *p* = 0.37). Finally, at p.e., SV response was more attenuated in LQT1 patients (ΔSV: +2 ± 0.5 vs. +4 ± 1%, *p* < 0.0001), but at recovery it was more attenuated in LQT2 patients (ΔSV: 3 ± 2 vs. −1 ± 2%, *p* < 0.0001).

### 3.8. Measurements Reproducibility

For all phases, there was good inter-observer agreement for the SD-tGLS, tGLS, tE_SR_, and FT indices at 0.969, 0.978, 0.984, and 0.98, respectively. The intra-observer agreement for these parameters was also good at 0.975, 0.986, 0.987, and 0. 98, respectively.

## 4. Discussion

### 4.1. Findings

Our findings showed that LQTS mutation carriers had a prolonged QTc interval, which correlated with prolonged and markedly dispersed myocardial contraction, delayed early relaxation phase, and decreased LV filling duration. Unlike controls, these electromechanical disturbances deteriorated further with exercise, during which additional decrease of the LV diastolic myocardial function and attenuated LV stroke volume response were noted. Such abnormal responses to exercise were of a greater degree in symptomatic patients and in the LQT1 subgroup and appeared to improve with B-blocker therapy. Worsening myocardial contraction dispersion (SD-tGLS) at p.e. was the strongest discriminator for previous clinical events. To the best of our knowledge, this is the first study that examines the relationship between repolarization, mechanical dyssynchrony, and diastolic function during dynamic exercise and the potential haemodynamic consequences in genetically confirmed symptomatic and asymptomatic LQTS patients.

### 4.2. Data Interpretation

#### 4.2.1. Electromechanical Response to Exercise

In LQTS subjects and opposite to that seen in controls, the already prolonged QTc interval did not shorten but increased further with exercise. Such electrical disturbances were related to mechanical changes; QTc correlated with the degree of mechanical contraction dispersion (SD-tGLS) and the duration of myocardial contraction (tGLS). These were also increased at rest and worsened during exercise in LQTS patients.

Such myocardial contraction disturbances may potentially affect diastolic function and LV filling at rest and to a greater extent during exercise [13]. In our study, LV myocardial contraction duration (tGLS) and dispersion (SD-tGLS) correlated directly with the tE_SR_ interval. This was longer in LQTS patients, indicating significant delay of the early relaxation phase within the cardiac cycle. The degree of this delay increased further during exercise in patients. Moreover, tE_SR_ correlated inversely with LV filling time, which was shorter and shortened further with exercise in LQTS patients.

However, shortening of LV filling duration during exercise may adversely affect the LV filling volume [10]. Indeed, attenuated SV response to exercise was noted in our LQTS patients. Decreased and dis-coordinated diastolic function may have also contributed to this. LQTS patients had lower longitudinal early diastolic SR, which did not increase during stress as it did in the controls. Early relaxation myocardial velocity was also delayed with respect to peak inflow velocity [Δt (E-E_SR_)], suggesting significant dis-coordination between LV filling and myocardial relaxation. These may also be reflected by the lower transmitral flow velocities and the higher E/e’ noted in LQTS patients during exercise.

Of particular interest is the relationship between the above mechanical disturbances and the development of symptoms in this group of patients. Dispersion of myocardial contraction and delayed early relaxation, especially during stress, were shown to be the stronger discriminators of previous clinical events, even stronger than QTc interval. LQTS patients usually develop symptoms with stress, at the time when all systolic and diastolic disturbances were shown in our patients to worsen and when SV response was also attenuated. However, an amplified SV response during stress states may also be associated with reduced and insufficient subendocardial flow [13]. This may be suggested by the finding of reduced LV longitudinal strain, which reflects subendocardial function [3,10]. Taking into consideration that the subendocardial layer encompasses the purkinje fibre conduction system, reduced subendocardial flow may play an additional role in arrhythmogenesis risk [13].

#### 4.2.2. Effects of B-Blocker Therapy

B-blockers are used in the management of LQT1 and LQT2 patients in an effort to reduce the incidence of cardiac events (14). They decrease sympathetic over stimulation and shorten the QTc interval [14]. They have also been suggested to improve the EM coupling response to exercise [4]. In our study, despite no differences in the QTc interval between treated and untreated patients, those on B-blocker therapy had less pronounced mechanical dispersion and contraction duration, less delayed early relaxation, and longer LV filling time at rest, which was more pronounced during exercise. Moreover, patients not on B-blockers had more attenuated SV response to exercise, despite similar SV values at rest. These results may point towards the beneficial effect of B-blockers in LQTS patients with significant electromechanical disturbances.

#### 4.2.3. LQTS Genotype and Exercise Response

In our study, LQT1 and LQT2 subgroups showed different patterns of EM response to exercise. QTc was prolonged and myocardial contraction dispersion was exaggerated at p.e. more so in the LQT1 than LQT2 patients. Such differences in EM response to exercise may reflect the different effects of adrenergic stimulation triggers in the two genotypes [15]. In LQT1, the genetic mutations drive changes in the slowly activating delayed rectifier potassium current (IKs) with subsequent effects in repolarization during exercise [16]. Therefore, inadequate action potential shortening and increased myocardial inotropy and lucinotropy during exercise may explain the more pronounced EM disturbances during stress in this group. On the other hand, in LQT2, defects relate to the rapidly activating component of the delayed rectifier potassium current (IKr), which affect repolarization at rest [16]. This may be responsible for the more intense changes noted during recovery in our LQT2 patients.

### 4.3. Clinical Implications

Identifying LQTS patients who are at risk for developing arrhythmia remains a challenge. In our study, EM disturbances were shown to be more pronounced in symptomatic LQTS mutation carriers, especially during exercise. In fact, myocardial contraction dispersion during exercise was the strongest discriminator of previous clinical events, over and above QTc. Such abnormal EM response to exercise was less pronounced in patients receiving B-blocker therapy. Therefore, additional assessment of EM response to exercise may contribute towards identifying LQTS mutation carriers at risk of arrhythmias and may play a role when deciding optimal management strategy.

### 4.4. Study Limitations

A modest number of LQTS subjects were included in this study and our results need to be reproduced in larger cohorts of carriers with better representation of genotype subgroups. Although between controls and patients there was no significant difference in peak exercise heart rate, this value was below that predicted for age in both groups. Lower achieved heartrate could be explained by decreased fitness levels in controls whereas B-blockers may be responsible for the attenuated heart rate response in patients. Cardiac events were identified based on the previously documented history.

## 5. Conclusions

LQTS carriers present marked LV electromechanical dispersion, which is associated with impaired longitudinal systolic and diastolic function and inadequate stroke volume response during exercise. These disturbances are more pronounced in patients with previous arrhythmic events. Assessment of LV myocardial dispersion and relaxation properties using myocardial deformation imaging techniques may be of additional value in risk stratification and management of LQTS carriers.

## Figures and Tables

**Table 1:** Left ventricular function at rest, peak exercise, and four minutes into recovery in LQTS.

	Rest			Peak			Recovery		
	LQT	Control	*p* Value	LQT	Control	*p* Value	LQT	Control	*p* Value
**QTc,** ms	453 ± 42	413 ± 17	<0.0001	499 ± 45	390 ± 19	0.0001	479 ± 35	414 ± 20	0.0001
*LV Contraction Parameters*									
**LV EF,** *%*	69 ± 4	70 ± 6	0.9	74 ± 5	78 ± 6	0.7	70 ± 3	72 ± 5	0.6
**GLS,** *%*	17 ± 4	19 ± 1.2	0.005	19 ± 3	23 ± 11	0.0001	17.1 ± 5	20 ± 2	0.0006
**tGLS**, *%*	47 ± 7	42 ± 6.3	0.0001	59 ± 10	40 ± 6	<0.0001	49 ± 6	40 ± 6	<0.0001
*LV Diastole Parameters*									
**E/A**	1.1 ± 0.13	1.2 ± 0.35	0.11	0.94 ± 0.2	1.2 ± 0.1	<0.03	1.12 ± 0.16	1.19 ± 0.24	0.15
**E DT,** ms	220 ± 58	169 ± 18	<0.0001	138 ± 42	101 ± 18	<0.0001	227 ± 81	170 ± 16	<0.0001
**E/E’ _lateral_**	9.2 ± 4.6	6 ± 1.2	0.0002	12.6 ± 5	6.3 ± 1.7	0.0026	9.3 ± 4.6	6.4 ± 1.31	<0.0001
**E/E’ _septal_**	12.4 ± 8	6.6 ± 1.30	0.0004	14.7 ± 9	6.5 ± 1.18	<0.0001	12.2 ± 8.4	6.9 ± 0.6	0.0005
**E_SR_**, s^−1^	1 ± 0.3	2 ± 0.3	<0.0001	1.1 ± 0.4	2.8 ± 0.4	<0.0001	0.95 ± 0.3	1.9 ± 0.5	<0.0001
**tE_SR_**, %	61 ± 7	58 ± 4	0.03	70 ± 6	56 ± 3	<0.0001	65 ± 7	57 ± 1	<0.0001
**FT,** *%*	44 ± 7	51 ± 0.9	<0.0001	34 ± 4	52 ± 2	<0.0001	41 ± 8	50 ± 5	<0.0001
*LV Mechanical Discoordination*									
**SD TGLS**, ms	58 ± 8	31 ± 8	<0.0001	61 ± 8	24 ± 10	< 0.0001	60 ± 10	26 ± 10	<0.0001
**t (E-E_SR_)**, ms	−8 ± 22	19 ± 7	<0.0001	−21 ± 25	17 ± 6	<0.0001	−9 ± 25	20 ± 6	<0.0001
*Myocardial Function Result*									
**SV**, mL	70 ± 6	69 ± 9	0.5	68 ± 10	96 ± 11	<0.0001	68 ± 8	71 ± 11	0.1

QTc: QT corrected, LV EF: left ventricular ejection function, GLS: global longitudinal systolic strain, tGLS: time-to-peak GLS, E/A: ratio of E wave over A wave, E DT: E-wave deceleration time, E/E’: ratio of E wave over E’, E_SR_: LV global longitudinal early diastolic strain rate, tE_SR:_ time to E_SR_, FT: filling time, SD TGLS: standard deviation of tGLS, t (E-E_SR_): time difference between tE and tE_S_, SV: stroke volume.

**Table 2:** Comparisons between symptomatic and asymptomatic LQTS mutation carriers.

	REST			PEAK			RECOVERY		
	Symptom.	Asymptom.	*p* Value	Symptom.	Asymptom.	*p* Value	Symptom.	Asymptom.	*p* Value
**HR**, b/min	65 ± 8	70 ± 14	0.1	121 ± 18	128 ± 20	0.2	76 ± 12	80 ± 16	0.3
**QTc,** ms	479 ± 43	447 ± 36	0.02	504 ± 41	479 ± 14	0.003	495 ± 39	469 ± 16	0.002
*LV Contraction Parameters*									
**GLS**,%	15 ± 2	19 ± 3	<0.0001	17 ± 5	21 ± 5	0.01	14.6 ± 3	18.4 ± 4	<0.0002
**tGLS**, *%*	52.3 ± 6.5	43 ± 5	<0.0001	64 ± 8.7	54.5 ± 8.8	0.0005	53.7 ± 6.7	45.8 ± 4	<0.0001
*LV Diastole Parameters*									
**E_SR_**, s^−1^	0.92 ± 0.23	1.12 ± 0.3	0.01	0.92 ± 0.23	1.26 ± 0.36	0.0006	0.75 ± 0.19	1.04 ± 0.25	0.0001
**tE_SR_**, *%*	64.58 ± 6.9	57.14 ± 3.6	0.0001	73.32 ± 4.3	67.1 ± 5.6	0.0002	69.75 ± 6.3	60.87 ± 60.4	<0.0001
**FT**, *%*	40 ± 3.2	47 ± 4.8	<0.0001	31 ± 4	39 ± 1.7	<0.0001	37.5 ± 4	44 ± 6	0.0001
*LV Mechanical Discoordination*									
**SD TGLS**, ms	62 ± 6	54 ± 8	0.0008	66 ± 8	54 ± 5	<0.0001	66 ± 8	56 ± 9	0.0004
**t(E-E_SR_),** ms	10 ± 5	33 ± 20	0.0007	18 ± 11	40 ± 24	0.003	13 ± 9	33 ± 28	0.0009
*Myocardial Function Result*									
**SV**, mL	63.8 ± 2.7	76 ± 6	<0.0001	65.9 ± 6.5	82 ± 7.7	<0.0001	61 ± 4	72 ± 10	<0.0001

HR: Heart rate, QTc: QT corrected, GLS: global longitudinal systolic strain, tGLS: time-to-peak GLS, E_SR_: LV global longitudinal early diastolic strain rate, tE_SR:_ time to E_SR_, FT: filling time, SD TGLS: standard deviation of tGLS, t (E-E_SR_): time difference between tE and tE_S_, SV: stroke volume.

**Table 3:** Comparisons between patients with LQTS treated with B-blockers and those that were untreated at rest, peak exercise, and four minutes into recovery.

	REST			PEAK			RECOVERY		
	B-Blockers	Untreated	*p* Value	B-Blocker	Untreated	*p* Value	B-Blocker	Untreated	*p* Value
**QTc,** ms	452 ± 46	454 ± 37	0.8	491 ± 37	509 ± 52	0.1	481 ± 38	477 ± 33	0.7
**GLS**, *%*	20 ± 8	16 ± 3	0.03	20 ± 4	16 ± 5	0.003	19 ± 8	16 ± 3	0.03
**tGLS**, *%*	43 ± 11	50 ± 6	0.01	54 ± 9	63.8 ± 8	0.0008	47 ± 6	50 ± 5	0.01
**SD TGLS**, ms	53 ± 7	63 ± 5	0.001	54 ± 3	65 ± 8	<0.0001	57 ± 10	64 ± 8	0.01
**E_SR_**, s^−1^	1.45 ± 1.2	0.8 ± 0.3	0.02	1.23 ± 0.3	0.99 ± 0.3	0.009	1.35 ± 1	0.75 ± 0.25	0.01
**tE_SR_**, *%*	57 ± 6	65 ± 6	0.002	67 ± 4	73 ± 6	0.0002	58 ± 6	65 ± 9	0.002
**FT**, *%*	47.5 ± 6	40 ± 6	0.003	35 ± 4	31 ± 3	<0.0004	47 ± 6	40 ± 6	0.003
**SV**, mL	75 ± 13	76 ± 15	0.8	73 ± 15	60 ± 17	<0.01	76 ± 18	63 ± 17	0.01

QTc: QT corrected, GLS: global longitudinal systolic strain, tGLS: time-to-peak GLS, FT: filling time, SD TGLS: standard deviation of tGLS, E_SR_: LV global longitudinal early diastolic strain rate, tE_SR:_ time to E_SR_, SV: stroke volume.

## Data Availability

The data presented in this study are available on request from the corresponding author. The data are not publicly available due to ongoing research analysis.
